# DWEC-YOLO: A Multi-Behavior Detection Model for Group-Housed Pigs

**DOI:** 10.3390/ani16193125

**Published:** 2026-10-05

**Authors:** Yan Chen, Jiehao Wu, Yingjie Kuang, Qiumei Yang

**Affiliations:** College of Mathematics and Informatics, South China Agricultural University, Guangzhou 510642, China; cheny@scau.edu.cn (Y.C.); kuangyj@scau.edu.cn (Y.K.); yqmbegonia@163.com (Q.Y.)

**Keywords:** pig behavior recognition, aggressive behavior, YOLO11, multi-behavior detection, smart livestock farming

## Abstract

In large-scale pig farming systems, high visual similarity among pigs, complex posture variations, and frequent interactions between individuals pose significant challenges to vision-based multi-behavior detection. To overcome these limitations, this study proposes an improved YOLO11-based pig behavior detection model, termed DWEC-YOLO, for recognizing normal behaviors (standing, lying, sitting, and feeding) and aggressive behaviors (fighting, ear biting, and tail biting). First, the C3k2-DySnake module is embedded within the backbone architecture, enabling more effective extraction of fine-grained visual details. Second, an EUCB-SC module is employed during feature aggregation and works jointly with the CoordAtt mechanism to strengthen multi-scale feature representations, thereby enhancing the network’s sensitivity toward critical interaction areas. In addition, Wise-ShapeIoU is adopted as the bounding-box regression loss to refine localization optimization and produce more accurate object predictions. Comprehensive experiments show that DWEC-YOLO surpasses several representative lightweight detectors, including YOLOv5n, YOLOv7-Tiny, YOLOv8n, YOLOv10n, and YOLOv12n, in detection effectiveness. Relative to the original YOLO11 architecture, DWEC-YOLO improves precision, recall, and mAP@0.5 by 4.28, 4.46, and 2.65 percentage points, respectively, achieving 96.17%, 95.25%, and 97.57%. These outcomes demonstrate that DWEC-YOLO substantially enhances multi-behavior detection in group-housed environments, serving as a dependable tool for automated monitoring in smart livestock farming.

## Simple Summary

Timely recognition of pig behaviors is essential for monitoring health status, detecting abnormal conditions, and improving management efficiency in modern pig farming. However, automatic multi-behavior recognition remains challenging due to high visual similarity among pigs, frequent movements, and mutual occlusions in group-housed environments. In response to these issues, a lightweight framework termed DWEC-YOLO (Dynamic Snake Convolution, Wise-ShapeIoU, EUCB-SC, and CoordAtt) was developed to recognize both normal and aggressive behaviors under complex farming conditions. Experimental data confirm that this framework successfully achieves stable, high-accuracy behavior detection under challenging environmental constraints, thereby providing a robust tool for intelligent pig farm management and supporting improved herd welfare and operational effectiveness.

## 1. Introduction

In modern livestock farming, as pig production continues to shift toward intensive and large-scale operations, pig health management and welfare tracking have become key priorities worldwide [1,2]. Pig behavior directly reflects their physiological status and environmental adaptability, serving as an important indicator for assessing their health condition [3]. Different behavioral patterns can provide valuable insights into the physiological state and social interactions of pigs [4]. Specifically, normal behaviors, such as standing, lying, and feeding, reflect basic physiological needs, while aggressive activities, such as fighting, ear biting, and tail biting, are associated with stress responses, social competition, and health risks [3,5,6,7]. Under high-density farming conditions, aggressive behaviors can occur suddenly and often involve multiple pigs, thus making the timely identification of such incidents crucial to prevent injuries, disease spread, and production losses [8,9,10]. In the long run, reliable identification of normal and aggressive pig behaviors is essential for efficient farm management and animal welfare [11]. To meet this demand, various monitoring approaches have been explored, ranging from manual observation to automated methods.

Traditionally, pig behavior monitoring mainly depends on human observation. Nevertheless, such manual approaches are constrained by heavy labor demands, substantial time investment, and inevitable subjective bias, rendering them unsuitable for long-term continuous monitoring [12]. Driven by advances in sensor technologies and artificial intelligence, automated behavior recognition methods have attracted increasing attention in recent years. Depending on the source of behavioral information, these automated strategies broadly fall into two categories: contact-based and non-contact-based solutions [13,14].

Contact-based behavior recognition frameworks generally depend on animal-mounted sensors to collect acceleration, posture, or physiological signals [15,16]. For instance, Lin et al. [17] proposed a hierarchical pig behavior recognition system based on electronic ear tags with triaxial accelerometers. The system employed logistic regression and support vector machines for behavior classification, achieving an average accuracy of 92%. In another study, Yin et al. [18] developed a wearable system for sheep heat stress detection, using LSTM and XGBoost for classification and achieving accuracies of 99% and 97.5%, respectively. Although contact-based methods can provide continuous and fine-grained temporal information, their practical application is limited by device-induced stress, high costs, and poor scalability in large-scale farming systems [19,20].

Non-contact behavior recognition methods typically use video analysis and deep learning models for behavior classification [21]. Specifically, Chen et al. [22] proposed a pig feeding behavior recognition method combining Xception and LSTM, in which spatial features were extracted and temporal information was modeled to improve recognition accuracy. Similarly, Zhang et al. [23] developed a spatiotemporal feature extraction model based on the SlowFast network for dairy goat behavior recognition, where performance was improved by enhancing the representation of key motion regions. These studies demonstrate that deep learning-based vision methods enable continuous monitoring without interfering with natural animal behavior and provide good real-time performance and strong scalability [24].

In large-scale farming environments, accurately distinguishing normal and aggressive behaviors in pigs is critical for ensuring animal welfare and reducing economic losses [25]. If not addressed promptly, aggressive behaviors may result in injuries among pigs and lower production efficiency, ultimately affecting farm profitability and management outcomes [26,27]. Therefore, accurate and automated pig behavior recognition in complex farming environments has become increasingly necessary for modern pig farming applications [28].

Recent developments in non-contact behavior recognition have advanced continuous and real-time observation of pig activities [29]. By eliminating the need for animal-mounted sensors, vision-based monitoring can capture group-level dynamic information without disrupting natural behaviors [30]. Consequently, this approach closely aligns with the demands of large-scale industrial farming and has swiftly moved into mainstream animal research [31]. Among the various vision-based solutions available, the YOLO series plays an important role due to its simple architecture, high detection speed, and strong accuracy. These merits enable real-time behavior recognition in complex multi-object scenarios, thereby leading to its widespread application in animal behavior monitoring [21]. A growing body of research has verified the applicability of YOLO-based approaches to pig behavior analysis. For example, Tu et al. [28] proposed an individual pig behavior tracking method based on YOLOv5-Byte, which could identify routine behaviors such as lying, standing, and feeding, achieving a mean Average Precision (mAP) of 92.9%. Huang et al. [32] developed a group-housed pig behavior tracking method based on an improved YOLOv8 and Deep OC-SORT, enabling effective recognition of behaviors including lying, standing, feeding, and drinking, with an mAP@0.5 of 98.9%.

While YOLO-based vision methods have achieved promising results in pig behavior recognition, several limitations still remain in existing studies. First, most existing studies focus primarily on common routine behaviors, whereas aggressive behaviors remain relatively underexplored despite their importance for early warning and farm management [33]. Second, in real large-scale farming environments, feature extraction is severely compromised by the high visual similarity among pigs, frequent occlusions, and complex interactions, thus hindering accurate behavioral differentiation [34]. To address these persistent challenges in precision livestock farming, we develop DWEC-YOLO, an upgraded YOLO11 variant tailored for pig multi-behavior recognition. The major contributions of this work are as follows:

- We propose a lightweight and accurate model, DWEC-YOLO, for recognizing both normal and aggressive behaviors in group-housed pigs. The model enhances behavior detection in complex interaction scenarios without sacrificing real-time performance.
- Dynamic Snake Convolution is integrated into the C3k2 structure to construct the C3k2-DySnake module, enhancing the representation of posture variations and complex deformation features, thereby improving the recognition of complex pig behaviors.
- An EUCB-SC feature fusion module is employed to effectively combine deep semantic features with shallow detailed information, enhancing the representation of behavioral targets across different scales.
- The CoordAtt attention mechanism is incorporated to emphasize key body regions and interaction areas of pigs, reducing interference from complex backgrounds and irrelevant information.
- The Wise-ShapeIoU loss function is utilized to refine the bounding-box regression process, improving localization precision and robustness in the presence of occlusion.

## 2. Materials and Methods

### 2.1. Data Sources and Preparation

The present study utilized a combined dataset comprising a public pig behavior recognition dataset and a self-collected dataset obtained from a commercial pig farm operated by Wens Foodstuff Group in Yunfu, Guangdong Province, China. The integration of public and farm-collected data enhanced the diversity of behavioral samples and farming scenarios, thereby improving the model’s adaptability to complex farming scenarios. For the self-collected portion, a total of 30 video recording sessions were conducted at the commercial pig farm, with each session lasting 40 min. Video footage was recorded using a Hikvision network camera (DS-2CD2047WD-L; Hangzhou Hikvision Digital Technology Co., Ltd., Hangzhou, China) operating at a 1920 × 1080 resolution and 25 FPS. The cameras were mounted at a height of approximately 2.8 m above the pens at a downward angle of roughly 80°. Recordings were conducted during daytime hours (08:00–18:00) under natural and auxiliary lighting. To reduce frame redundancy while capturing distinct behavioral transitions, video frames were sampled at a uniform interval of 1 frame every 5 s (a 1:125 frame-sampling ratio).

**Figure 1:**
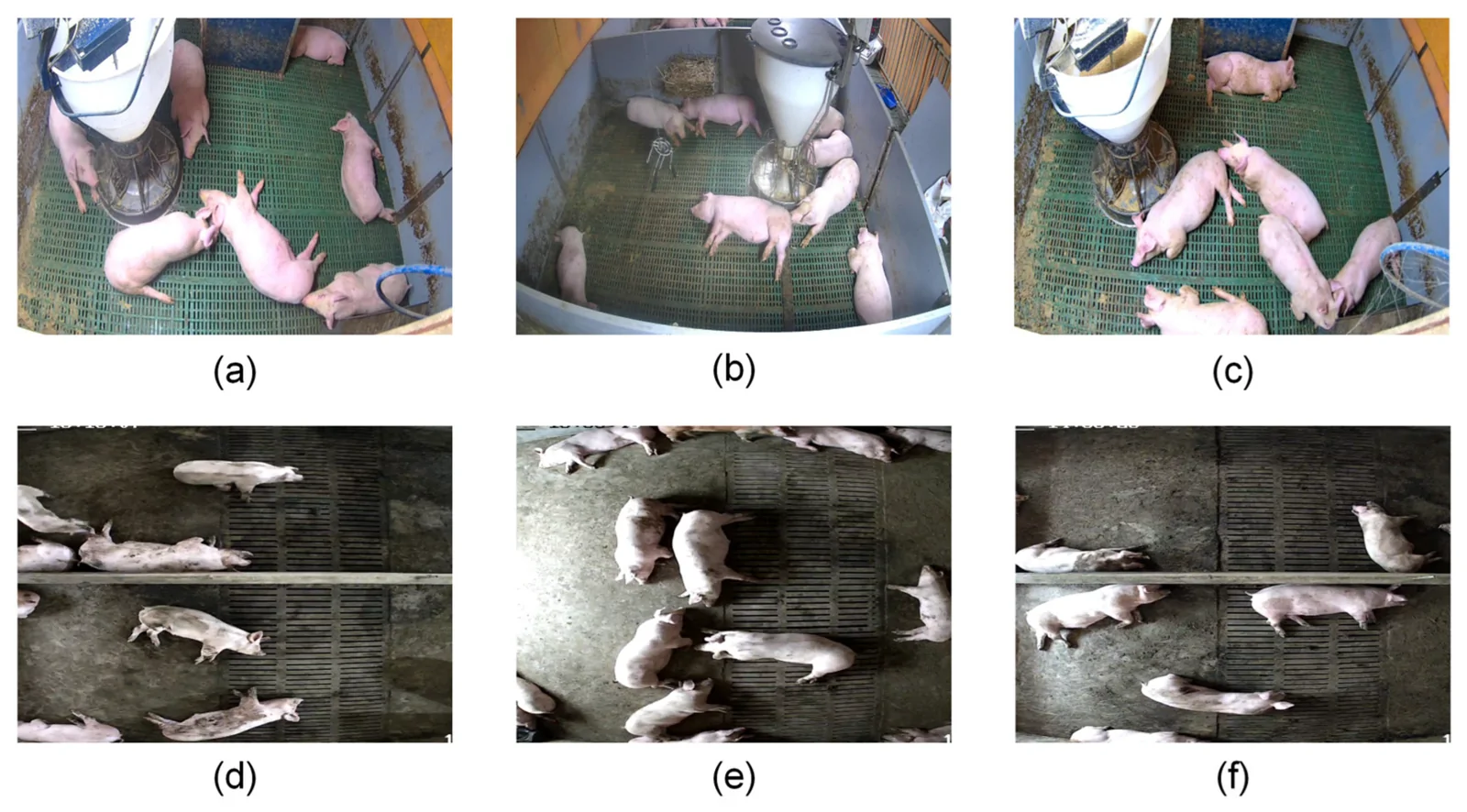
Representative samples from the datasets: (**a**–**c**) samples from the public dataset; (**d**–**f**) samples from the self-collected dataset.

Figure 1 shows representative samples from the dataset, where Figure 1a–c are from the public dataset and Figure 1d–f are from the self-collected dataset.

A total of 2205 images were included in the dataset, comprising 1358 images from the public dataset and 847 images from the self-collected dataset. The dataset was divided into training, validation, and testing sets at a ratio of 7:2:1. For the self-collected dataset, images were partitioned at the recording-session level, with all frames from the same uninterrupted recording assigned to a single subset to prevent temporally adjacent frames from being distributed across different subsets. The public dataset did not provide sufficient recording-session information to support the same session-level partitioning. Therefore, the public images were manually screened before splitting to remove duplicated or highly similar images and thereby reduce the potential for information leakage. The training subset was used for model optimization, while the validation subset was used for model selection and training monitoring. The testing set was kept independent of model development and used only for the final performance evaluation. The distribution of behavior categories across the three subsets, further separated by data source, is presented in Table 1.

All images were annotated manually using the LabelImg tool, and the resulting labels were saved in YOLO-format TXT files. The annotation followed the Pascal VOC standard [35]. During annotation, the degree of body occlusion was visually estimated by assessing the proportion of the animal’s body that was obscured from view, and individuals with more than approximately 75% of their body occluded were not annotated. For partially occluded individuals, bounding boxes were drawn around the visible parts of the animal only, without inferring the locations of occluded body parts. Seven pig behavior categories were defined in this study, including normal behaviors (feeding, standing, sitting, and lying) and aggressive behaviors (fighting, ear biting, and tail biting). Each bounding box was assigned only one behavioral label. No predefined minimum duration threshold was applied, and each image was annotated independently based on the clearly observable behavior in that frame. When a posture category (Stand, Sit, or Lie) occurred simultaneously with another behavior (Feeding, Fighting, Ear biting, or Tail biting), the latter was selected as the label. For example, a pig standing while feeding was labeled as Feeding rather than Stand, while a pig standing while tail biting was labeled as Tail biting rather than Stand. For ear biting and tail biting, the bounding box was annotated around the behavior-performing pig while including the key interaction cues. Accordingly, the reported metrics reflect the detection performance of the predefined behavior categories, including both individual behaviors and interaction behaviors. The definitions of each behavior category and representative example images are provided in Table 2.

### 2.2. Data Augmentation

To build a more resilient model and mitigate the potential effects of imbalanced data, data augmentation was introduced to increase the diversity of the training samples. Table 1 presents the original dataset distribution across the training, validation, and test sets. Following augmentation, the training set increased from 1543 to 3810 images, while the validation and test sets remained unchanged. The augmentation operations comprised random HSV color adjustments, horizontal flipping, Gaussian noise injection, small-angle rotation, and random brightness and contrast variation. No additional class-weighting, class-balanced loss, or focal loss variant was introduced in the training process. This augmentation-based strategy was used to alleviate the potential effects of uneven class distributions while maintaining the original detection loss formulation. The approach also helps stabilize the training process and reduce the risk of overfitting.

In detail, photometric augmentations, including random HSV color space adjustments together with brightness and contrast variations, were employed to replicate diverse illumination conditions and local shadows. Geometric augmentations, including horizontal flipping and small-angle random rotations, were further introduced to improve robustness to changes in movement direction, posture, and camera viewpoint. Furthermore, Gaussian noise injection was applied to mimic potential sensor interference during image acquisition. Together, these complementary augmentation strategies enriched sample diversity, ultimately improving the recognition performance for both normal and aggressive pig behaviors.

### 2.3. YOLO11 Backbone Network

As a member of the YOLO series, YOLO11 is an optimized object detection model with improvements in its architecture design [36]. Owing to its balance between accuracy and inference efficiency, YOLO11 was selected as the baseline model for this study to support our targeted behavior recognition tasks.

**Figure 2:**
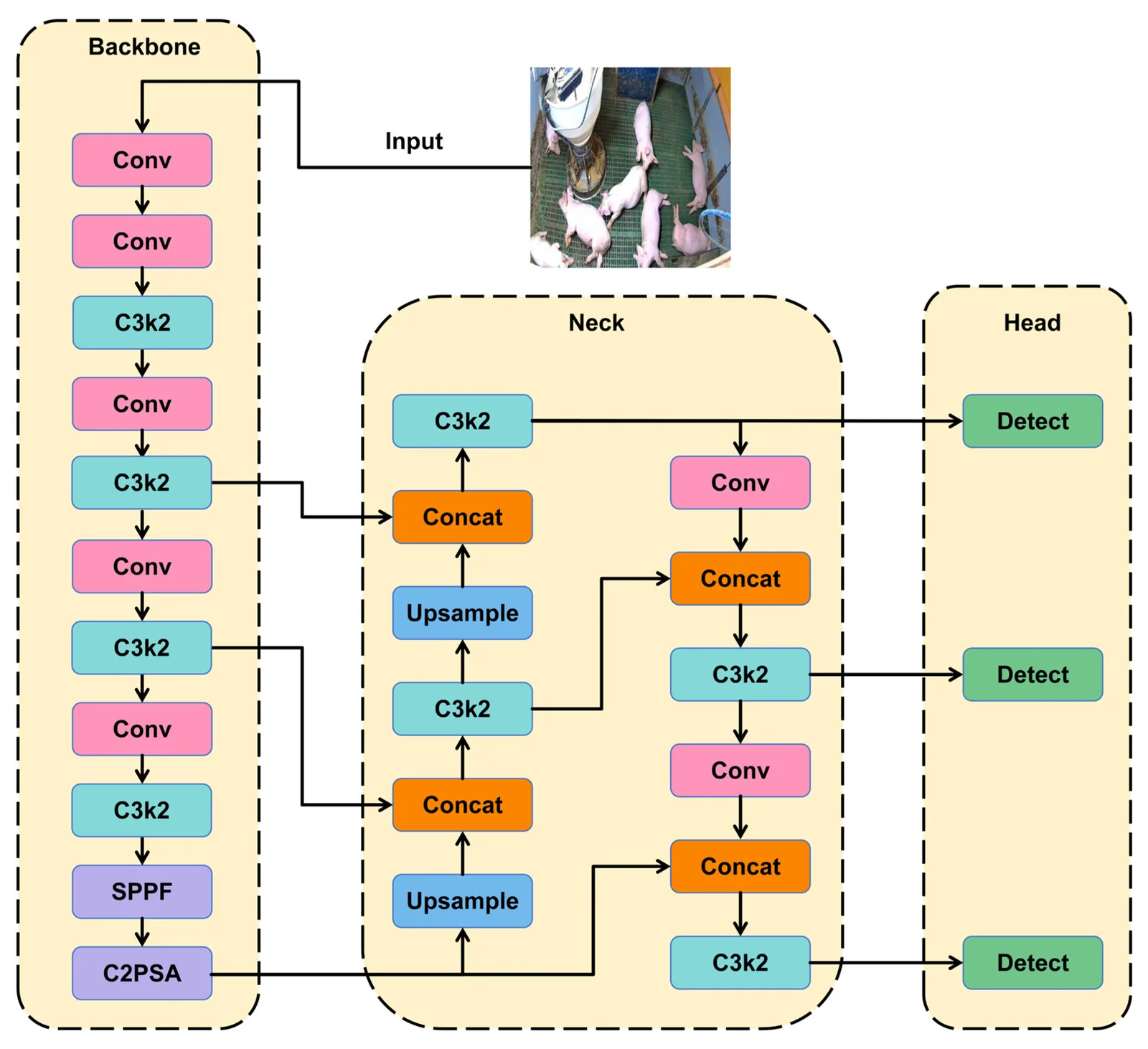
Structure of YOLO11 model.

As shown in Figure 2, the YOLO11 architecture is composed of a backbone for feature extraction, a neck for multi-scale feature fusion, and a detection head for classification and localization.

Among its components, several key modules are designed to improve feature extraction and representation learning. The backbone integrates the C3k2 block to achieve lightweight yet high-performance feature extraction while reducing computational overhead. Concurrently, positioning the SPPF module at the backbone’s termination facilitates receptive field enlargement and multi-scale pooling-driven contextual gathering. In addition, the C2PSA module enhances feature representations by directing heightened spatial-channel awareness toward highly discriminative regions.

Although YOLO11 has achieved strong performance in general object detection tasks, its original architecture is not specifically designed for pig behavior recognition. In practical farming environments, normal behaviors often exhibit high visual similarity, whereas aggressive behaviors such as fighting, ear biting, and tail biting are frequently associated with intensive interactions and occlusions. These challenges make accurate behavior recognition more difficult. To address this issue, we propose DWEC-YOLO, a pig behavior detection model based on YOLO11.

### 2.4. DWEC-YOLO Model Construction

**Figure 3:**
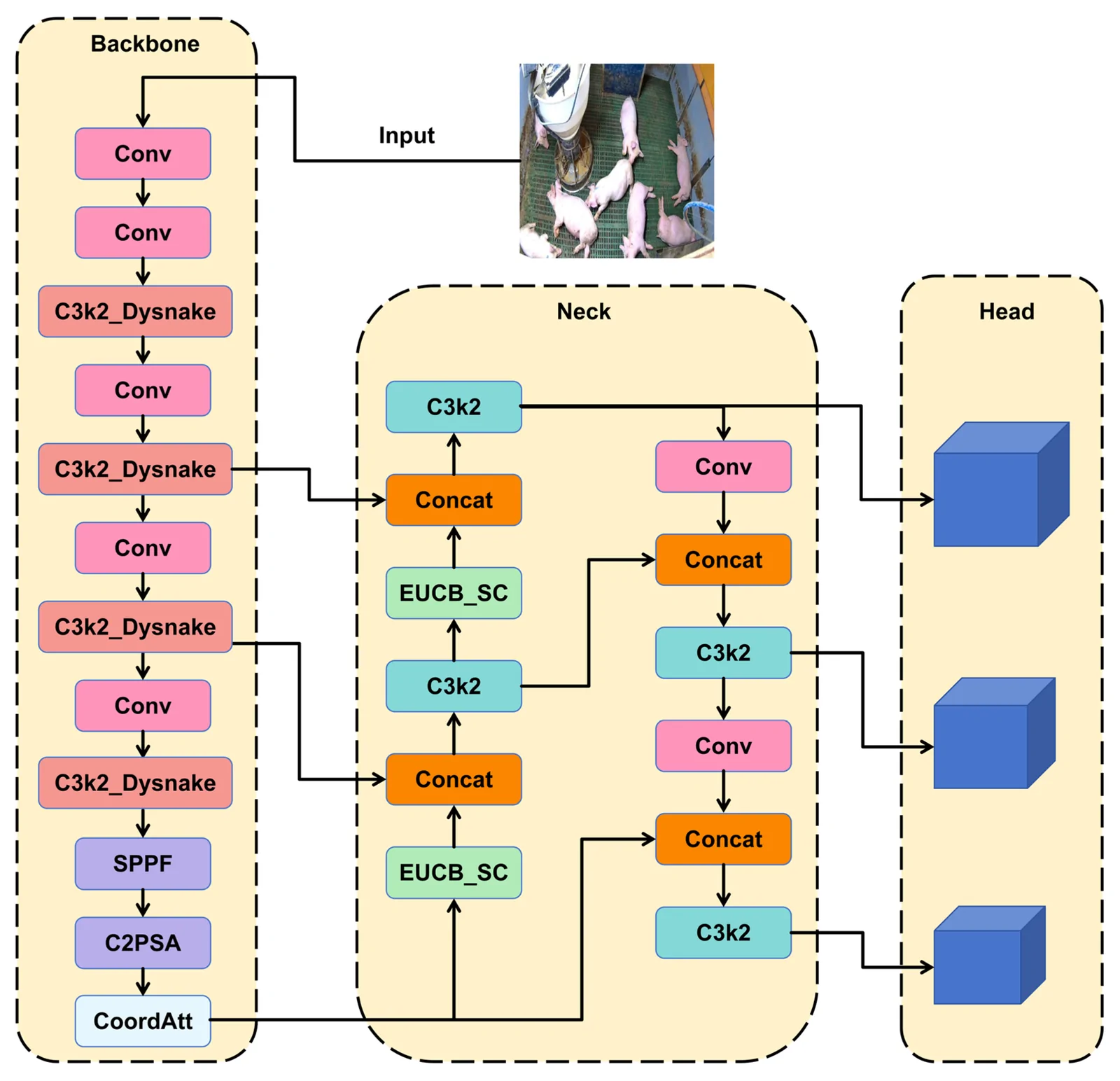
Structure of DWEC-YOLO model.

DWEC-YOLO is designed for pig behavior detection under group-housing conditions with large appearance variations and frequent occlusions. It incorporates several improvements to the backbone network, feature fusion structure, and bounding box regression strategy. The overall network architecture is shown in Figure 3.

Structurally, in terms of the backbone network, the standard feature extraction blocks are substituted with C3k2-DySnake modules. This introduction makes the framework much more sensitive to variations in pig body poses and fine-grained behavioral details. In the neck network, an EUCB-SC upsampling module is adopted to replace the original upsampling operation, improving information propagation during multi-scale feature fusion. In addition, a CoordAtt attention mechanism is incorporated to enhance the model’s focus on key behavioral regions while preserving positional information. Furthermore, to improve the bounding box regression process, Wise-ShapeIoU is used as the localization loss function, enabling better learning of object shape and geometric variations.

#### 2.4.1. C3k2-DySnake Module

For aggressive behaviors in pigs, discriminative information is mainly reflected in local limb movements and structural changes in key body parts. Specifically, these aggressive interactions are concentrated in the head, ear, and tail regions, characterized by close body contact, limb bending, and mutual deformations during pulling. Compounding these challenges in complex group-housed environments, frequent partial occlusion dictates that individual targets are often presented as fragmented structures rather than complete contours. This highlights the need for models to focus not only on local detailed features but also on spatial relationships among incomplete structures. The C3k2 module in YOLO11 relies on fixed convolution kernels for feature sampling, enabling effective representation of local texture and static appearance features. However, its receptive field is relatively regular, and fixed sampling locations limit its ability to adaptively model continuous structural relationships in complex local regions, which constrains its ability to capture fine-grained structural changes and interaction-related features under occlusion and pose variation.

To better adapt to these limitations, Dynamic Snake Convolution (DSConv) [37] is integrated into the C3k2 module, forming the proposed C3k2-DySnake block. By introducing learnable offsets, DSConv enables sampling locations to dynamically conform to the geometric distribution of the target, thereby shaping a responsive receptive field that aligns precisely with object contours. Compared with the fixed and regular sampling pattern of standard convolution, DSConv is more flexible in adjusting to changes in object structure and enhances the ability to model continuous spatial features.

To facilitate the description of the differences between standard convolution and the improved method, we first define the coordinate set *K* of the standard 2D convolution, where the center coordinate is denoted as *K_i_* = (*x_i_*, *y_i_*). For a 3 × 3 convolution kernel *K*, its sampling positions can be formulated as:

(1) K = {(x−1, y−1), (x−1, y), … , (x+1, y+1)}.

**Figure 4:**
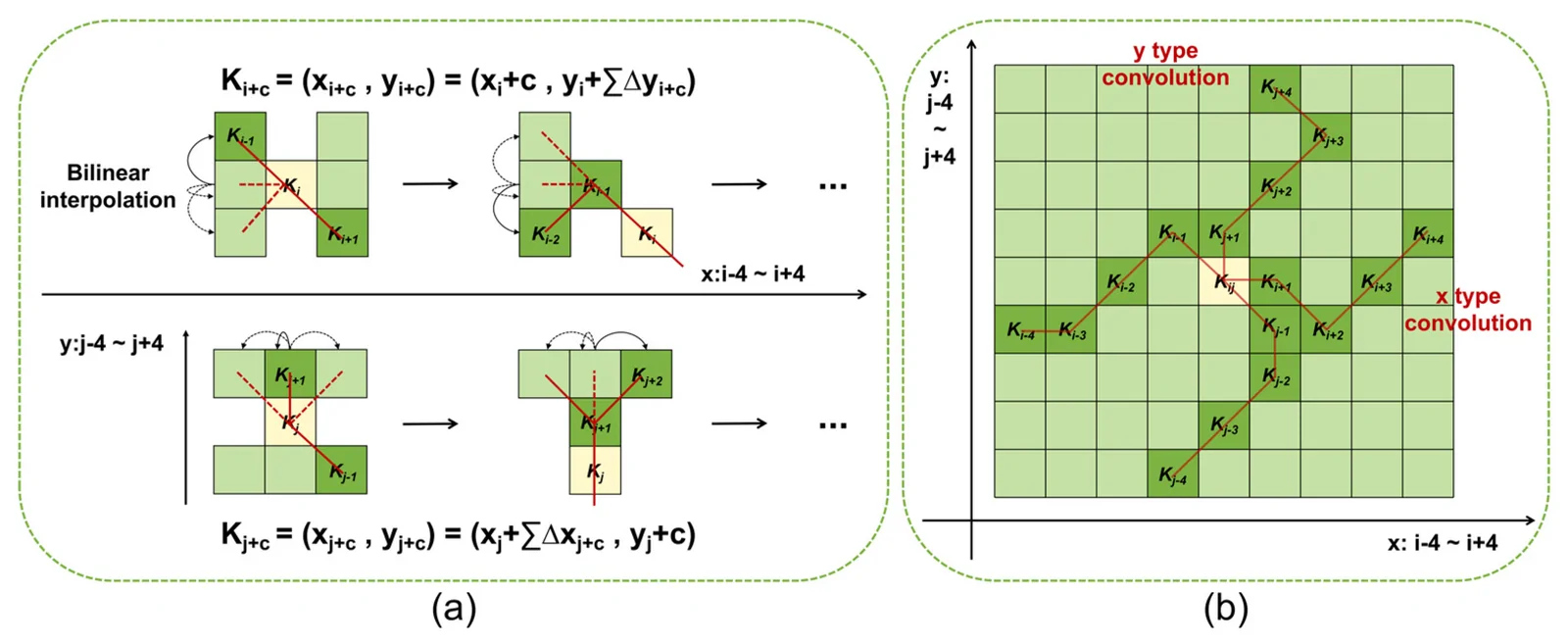
Coordinate computation and receptive field configurations of DSConv. (**a**) Coordinate computation process. (**b**) Optional receptive field configurations.

This formulation indicates that the receptive field of standard convolution consists of predefined discrete coordinates, which maintain a fixed spatial structure during feature extraction. Such a sampling mechanism is effective for objects with relatively stable shapes, but its adaptability is limited when handling targets with complex deformations or substantial structural variations. To address this limitation, deformable convolution introduces learnable offsets that enable adaptive sampling according to object shapes. However, because each sampling point is shifted independently, the resulting spatial distribution may become discontinuous, limiting the modeling of continuous structural patterns. To overcome this drawback, DSConv adopts a continuous recursive offset strategy, which progressively extends sampling locations along the object structure to form a snake-like dynamic receptive field. The coordinate computation process of DSConv and its optional receptive field configurations are illustrated in Figure 4.

In DSConv, the convolution kernel is first linearly extended along a single direction. Taking the x-direction as an example, its sampling locations can be expressed as:

(2) $$K_{i\pm c}\;=\;\left\{\begin{array}{c} (x_{i+c},y_{i+c})\;=\;\left(x_{i}+c,y_{i}+\sum_{i}^{i+c}\Delta y\right)\\ (x_{i-c},y_{i-c})\;=\;\left(x_{i}-c,y_{i}+\sum_{i}^{i-c}\Delta y\right) \end{array}\right.$$

Here, Δ*y* is the learnable offset of the convolution kernel in the vertical direction. In contrast to standard deformable convolution, which learns each offset independently, DSConv updates the sampling locations by accumulating the offsets step by step. This maintains continuity between adjacent sampling points, which in turn improves the modeling of the object structure’s orientation.

Similarly, when the convolution kernel is extended along the y-direction, its sampling locations can be expressed as:

(3) $$K_{j\pm c}\;=\;\left\{\begin{array}{c} (x_{j+c},y_{j+c})\;=\;\left(x_{j}+\sum_{k=j}^{j+c}\Delta x,y_{j}+c\right)\\ (x_{j-c},y_{j-c})\;=\;\left(x_{j}+\sum_{k=j}^{j-c}\Delta x,y_{j}-c\right) \end{array}\right.$$

Based on the above sampling strategy, DSConv can dynamically adjust the convolution kernel sampling path in both horizontal and vertical directions. This enables the receptive field to better adapt to variations in pig body contours.

**Figure 5:**
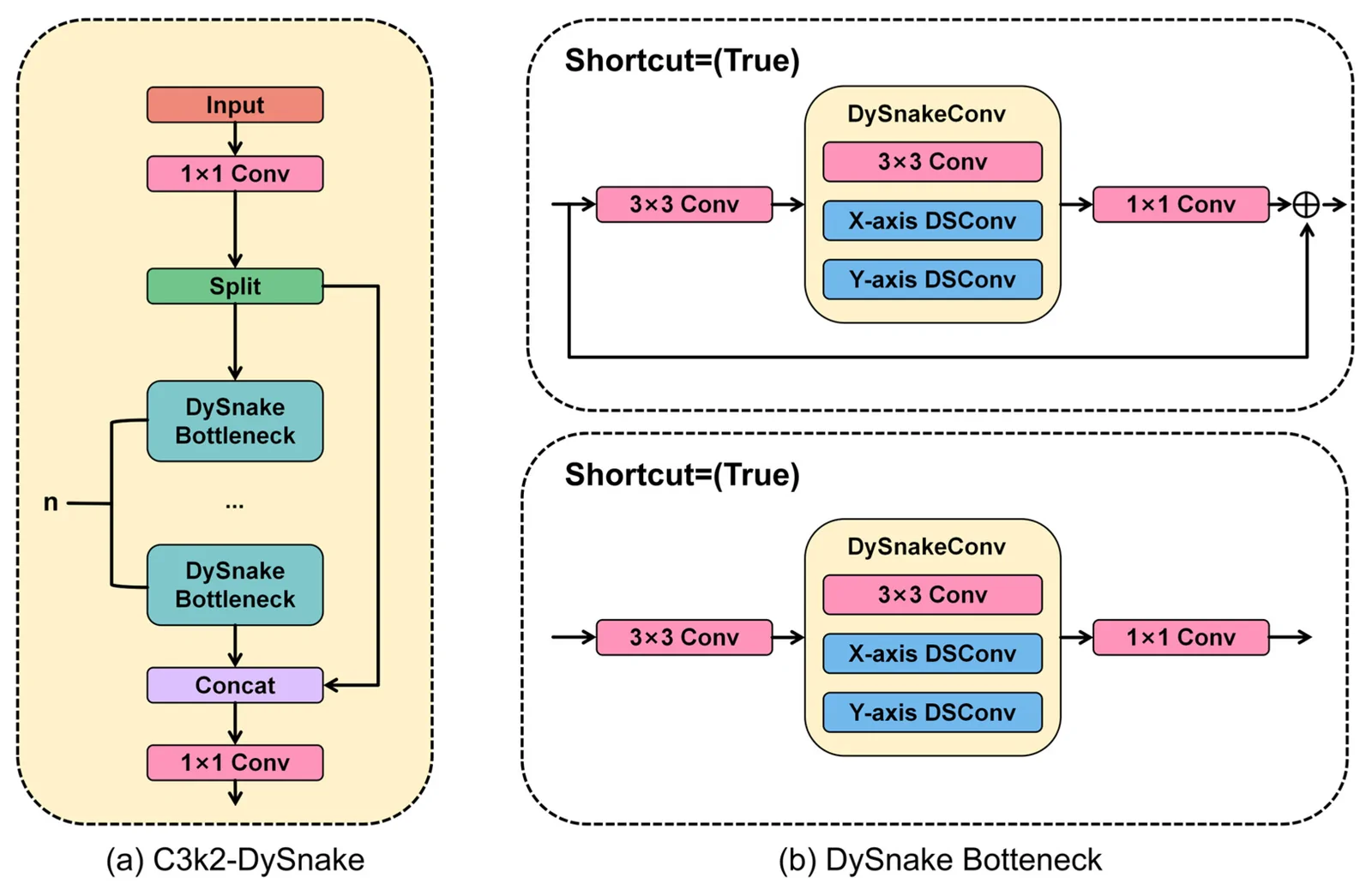
Structures of the C3k2-DySnake module and its bottleneck. (**a**) C3k2-DySnake structure. (**b**) DySnake Bottleneck structure.

The C3k2-DySnake module retains the overall architecture of the original C3k2 module while replacing its internal Bottleneck with a DySnake Bottleneck that incorporates DSConv for dynamic convolutional sampling. Figure 5a shows the overall structure of the C3k2-DySnake module, and Figure 5b presents the internal architecture of the DySnake Bottleneck.

As shown in Figure 5b, the input features undergo a 3 × 3 convolution to extract basic local features before entering the DySnakeConv module. Upon entering, the module utilizes X-axis DSConv and Y-axis DSConv to dynamically adjust the convolution kernel sampling locations horizontally and vertically. As a result, the receptive field can adaptively expand along the target structure, improving the extraction of features related to pig movements and body structures. In addition, the residual connection is retained to enhance feature reuse and maintain training stability.

By introducing DySnake Bottleneck, the improved C3k2-DySnake module enhances the representation of complex pose variations and local behavioral features, while successfully preserving the lightweight nature of the baseline network.

#### 2.4.2. EUCB-SC Upsampling Module

In pig behavior recognition, substantial variations exist in the scale distribution of different behavioral targets. Particularly in group-housed environments, factors such as imaging distance, individual occlusion, and posture variation cause behavioral regions to appear at different scales within images. Recognition of normal behaviors mainly depends on overall body posture and therefore requires relatively complete global visual information. In contrast, aggressive behaviors are often characterized by local interaction cues and their key discriminative information is typically represented by small-scale features, which makes them more sensitive to local details. During cross-scale feature fusion in the feature pyramid, information from small-scale regions may not be sufficiently enhanced. This can gradually weaken critical behavioral features during upsampling and fusion, making fine-grained behaviors more difficult to distinguish. In YOLO11, this issue is partly attributed to the limited cross-scale interaction capability of the original upsampling module. Detailed information from small-scale behavioral regions may not be effectively preserved during feature reconstruction, which hinders the coordination between deep semantic data and shallow visual details.

To improve multi-scale feature fusion, an EUCB-SC upsampling module was incorporated into the YOLO11 feature fusion stage as an enhanced version of the original EUCB [38]. By introducing the Shift Channel Mix mechanism [39], it strengthens information interaction across channels and improves information transfer among features at different scales.

**Figure 6:**
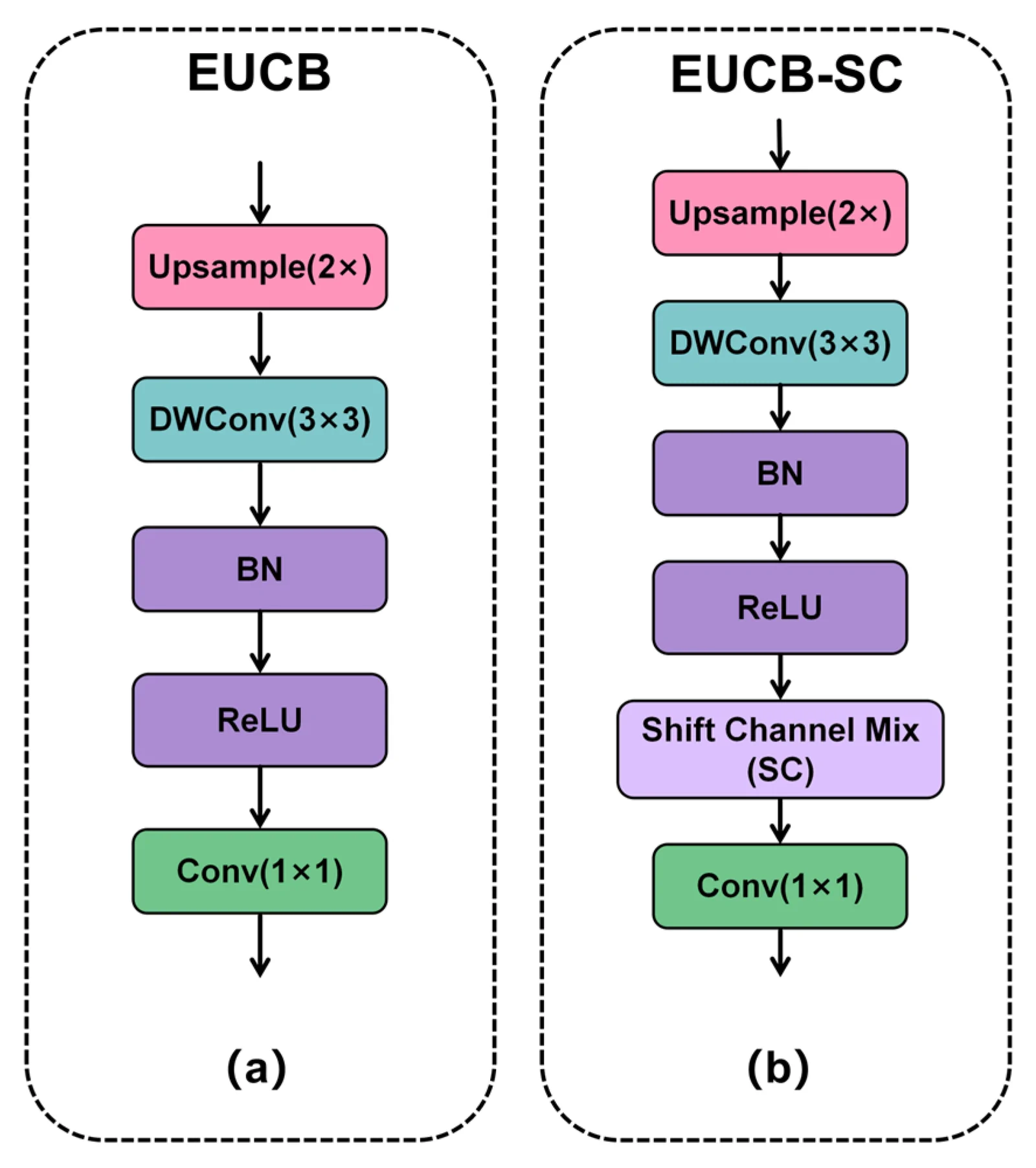
Structures of EUCB and EUCB-SC. (**a**) EUCB structure. (**b**) EUCB-SC structure.

As shown in Figure 6a, the original EUCB mainly consists of an Upsample layer, Depthwise Convolution (DWConv), Batch Normalization (BN), a ReLU activation function, and a 1 × 1 convolution. During execution, the Upsample layer first restores the spatial resolution of the feature map before passing it directly to the DWConv for efficient spatial feature extraction. After this, BN and ReLU are applied to stabilize training and enhance nonlinear feature representation. Finally, a 1 × 1 convolution is applied to fuse channel information.

While this standard configuration handles spatial features effectively, the original EUCB still has limitations in channel-wise information interaction, which may lead to insufficient fusion of features at different scales. To address this issue, the Shift Channel Mix (SC) module was further integrated into EUCB to construct the EUCB-SC structure. The overall architecture of the proposed module is illustrated in Figure 6b.

**Figure 7:**
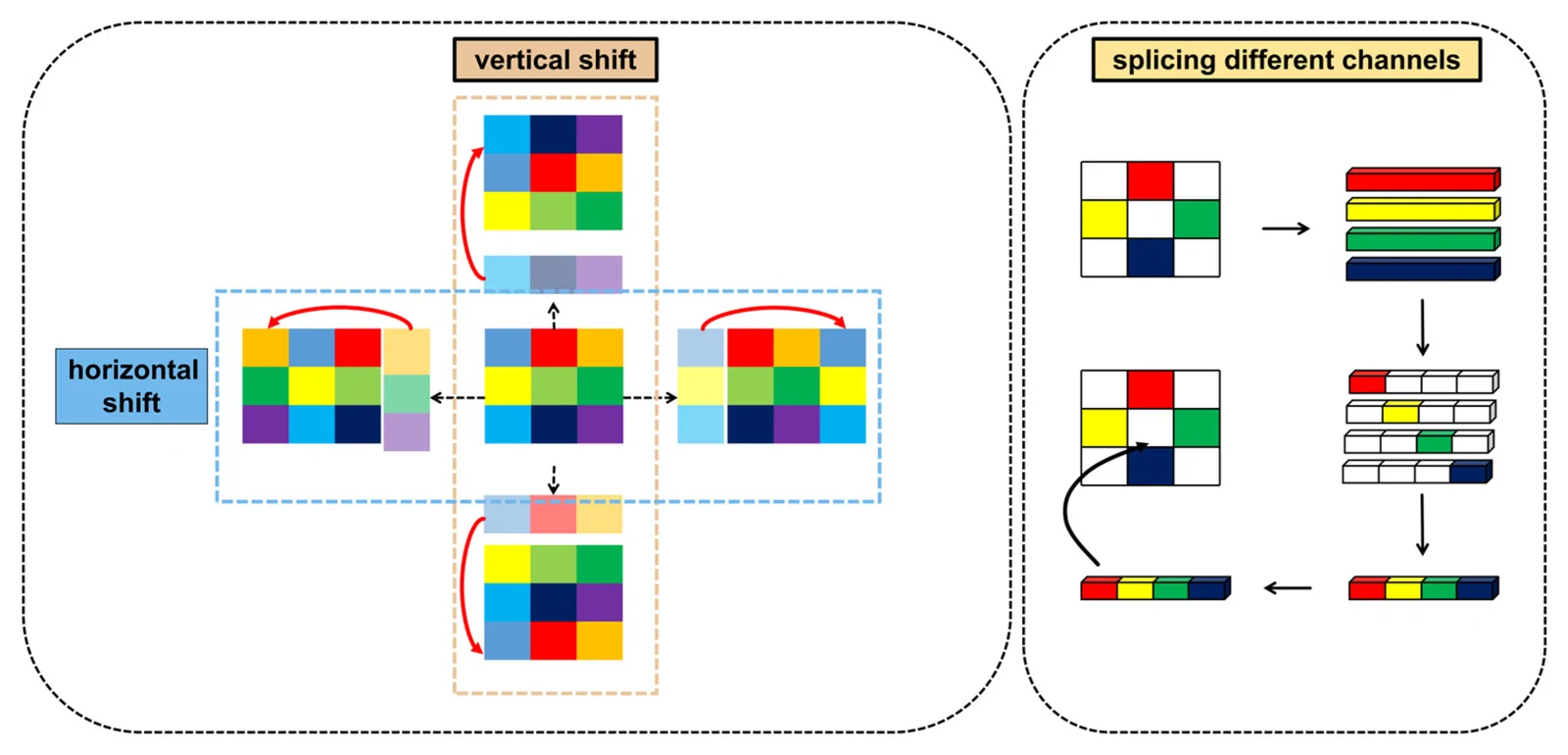
Workflow of the Shift Channel Mix (SC) module.

The SC module is a key component of EUCB-SC and is designed to enhance information interaction across channels. As illustrated in Figure 7, the module first applies horizontal or vertical spatial shifts to a portion of the channel features, enabling neighboring spatial information to be incorporated without introducing additional convolution operations. Subsequently, the shifted features are rearranged across channels, promoting information exchange among different feature groups and enhancing cross-channel interactions. Importantly, since both operations rely on feature manipulation rather than learnable convolution kernels, they impose only a negligible computational burden. Consequently, the module effectively strengthens feature representation across both spatial and channel dimensions without sacrificing computational efficiency.

In the group-housed pig behavior dataset used in this study, different behavioral targets exhibit distribution variations across feature layers of different scales. In addition, occlusion and variations in target distance further increase the difficulty of feature fusion, making behavioral information more susceptible to degradation during the fusion process. To mitigate these issues, EUCB-SC strengthens information exchange across feature scales and promotes more effective fusion of semantic and spatial features. This helps the model capture behavioral characteristics more effectively in complex group-housed environments.

#### 2.4.3. CoordAtt Integration

**Figure 8:**
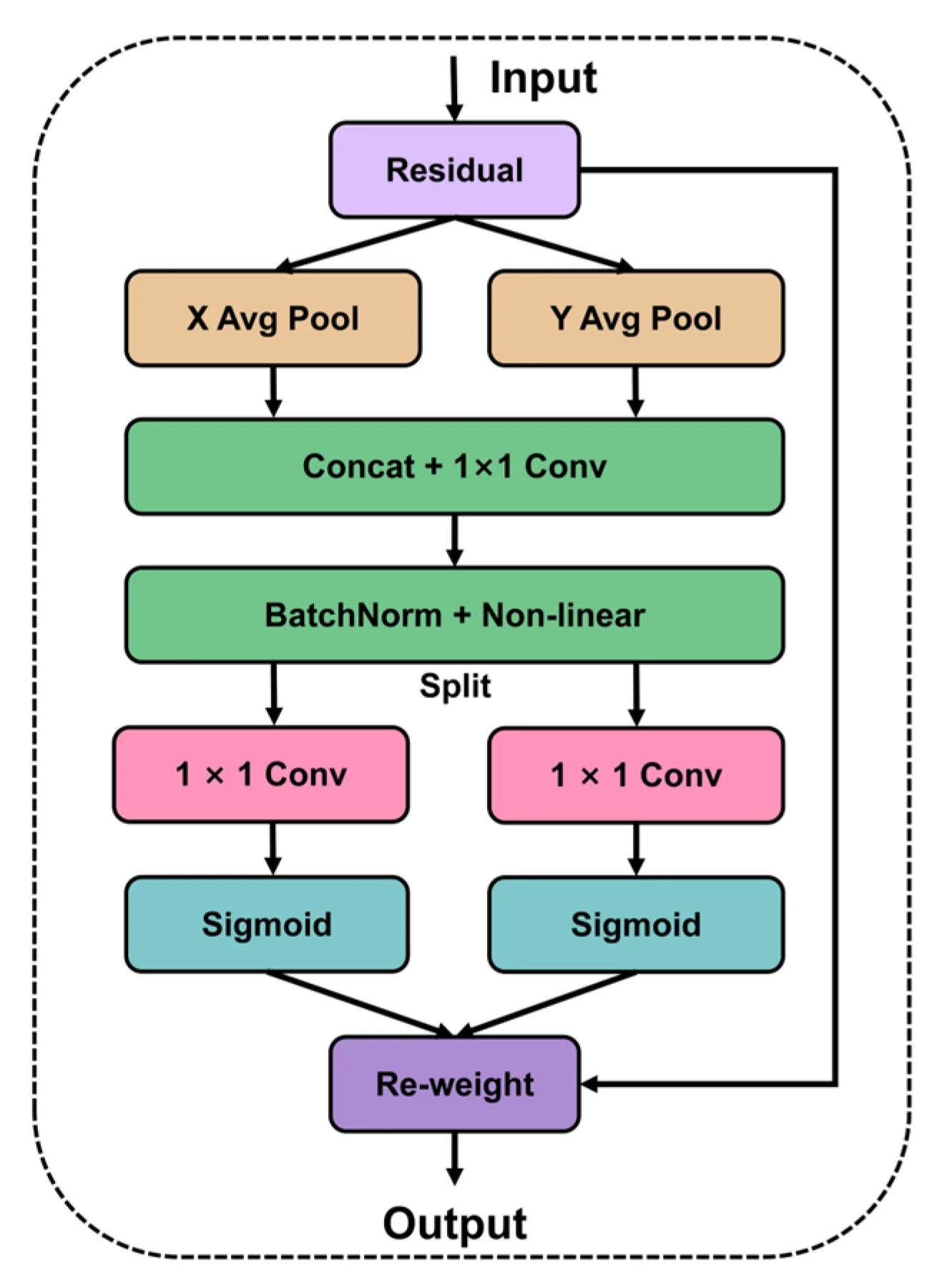
Coordinate attention mechanism.

To give the model a sharper focus on key behavioral regions, the CoordAtt attention mechanism [40] was integrated into the network. Its structure is shown in Figure 8. Unlike attention mechanisms that focus solely on channel-wise modeling, CoordAtt embeds positional information into feature encoding during channel attention computation. It achieves this by separately encoding spatial information along the horizontal and vertical axes for subsequent attention weight generation and feature modulation. This process facilitates a more precise focus on behavior-related regions, particularly key body parts and interaction areas, thereby improving behavior recognition and localization.

Specifically, CoordAtt first aggregates global information from the input feature map along the horizontal and vertical spatial directions. The aggregated features are compressed into two one-dimensional representations, which reduce spatial dimensions while preserving directional positional information. Next, a 1 × 1 convolution, Batch Normalization (BN), and a non-linear activation act upon the concatenated directional features to yield a combined intermediate representation. This newly formed representation is immediately divided into horizontal and vertical components, from which attention weights are generated for the corresponding directions. After Sigmoid normalization, the attention weights are applied to the input feature map through element-wise multiplication. As a result, the network can assign different importance to features at different spatial locations, thereby enhancing the localization of behavior-related regions.

This mechanism integrates spatial positional information into channel feature modeling, enabling the learned features to contain both semantic information and directional spatial cues. Furthermore, it helps the network better distinguish spatial response differences and enhances behavior-related structural representation. Overall, the introduction of CoordAtt sharpens the model’s perception and localization of key behavioral regions.

#### 2.4.4. Wise-ShapeIoU Loss Function

The bounding box regression loss is essential for steering the network toward precise object positions and shapes, which directly determines localization performance. Traditional IoU-based methods and their variants (e.g., GIoU [41], DIoU [42], and SIoU [43]) are centered on optimizing spatial overlap and geometric alignment relative to the true target boundaries, and they generally achieve satisfactory localization performance in most scenarios. However, their adaptability becomes limited when object shapes vary significantly. In pig behavior recognition tasks, a single target (an individual pig) may exhibit different behaviors such as standing, lying, and feeding. Due to posture changes, the aspect ratio and overall shape of the bounding box can vary considerably, making it difficult for traditional IoU constraints to fully reflect such variations.

In response to this limitation, ShapeIoU [44] is adopted in this work as the baseline regression loss. Compared with traditional IoU, ShapeIoU incorporates an additional shape constraint term, allowing the model to optimize not just the overlapping region, but specifically the predicted boxes’ structural consistency with their ground-truth counterparts throughout the optimization phase. Its formulation is given as follows:

(4) $$L_{shape-IoU}=1-IoU+{distance}_{shape}+0.5\times \Omega_{shape}$$

where *IoU* (the overlap measure) is defined as the intersection-over-union between the predicted bounding box B and the ground-truth box B_gt_:

(5) $$IoU=\frac{\left|B\cap B^{gt}\right|}{\left|B\cup B^{gt}\right|}$$

*distance_shape_* (geometric structural difference) is defined as:

(6) $${distance}_{shape}=hh\times \frac{{\left(x_{c}-x_{c}^{gt}\right)}^{2}}{c^{2}}+ww\times \frac{{\left(y_{c}-y_{c}^{gt}\right)}^{2}}{c^{2}}$$

where the respective center coordinates of the predicted and ground-truth bounding boxes are represented by $\left(x_{c},\;y_{c}\right)$ and $\left(x_{c}^{\mathrm{gt}},\;y_{c}^{\mathrm{gt}}\right)$. The variable *c* signifies the diagonal length of the smallest circumscribed rectangle, while *ww* and *hh* serve as directional weighting coefficients that are adaptively calculated from the width–height proportions of the ground-truth box:

(7) $$ww=\frac{2\times (w^{gt}{)}^{scale}}{(w^{gt}{)}^{scale}+(h^{gt}{)}^{scale}},\;hh=\frac{2\times (h^{gt}{)}^{scale}}{(w^{gt}{)}^{scale}+(h^{gt}{)}^{scale}}$$

*Ω_shape_* (shape penalty term) is used to further constrain differences in width–height structure:

(8) $$\Omega_{\mathrm{shape}}=\sum_{t\in \left\{w,h\right\}}{\left(1-e^{-\omega_{t}}\right)}^{\theta },\;\theta =4$$

where the weighting terms *ω_w_* and *ω_h_* are defined as:

(9) $$\left\{\begin{array}{c} \omega_{w}=\mathrm{hh}\times \frac{\left|w-w^{gt}\right|}{\max(w,w^{gt})}\\ \omega_{h}=\mathrm{ww}\times \frac{\left|h-h^{gt}\right|}{\max(h,h^{gt})} \end{array}\right.$$

To further improve the optimization ability of ShapeIoU on hard samples, this study introduces the Wise-IoU v3 strategy [45], which dynamically adjusts the gradient contributions across various instances based on a quality assessment factor, thereby reducing the influence of low-quality predictions during optimization. Finally, the Wise-ShapeIoU loss function is defined as:

(10) $$L_{Wise-ShapeIoU}=r\cdot \left(L_{Shape-IoU}\right)$$

where *r* is a gradient adjustment coefficient, defined as:

(11) $$r\;=\;\frac{\beta }{\delta \;\alpha^{\beta -\delta }}$$

*β* represents the regression difficulty coefficient of the current sample, which reflects the deviation of its ShapeIoU loss from the average loss within a batch. *α* and *δ* are hyperparameters and are set to 1.9 and 3, respectively. These parameters control the nonlinear mapping of sample weights and contribute to stable model training and convergence.

### 2.5. Experimental Environment and Parameter Settings

All training and evaluation processes for the proposed DWEC-YOLO were conducted utilizing a high-performance workstation. The hardware configuration included an Intel Core i9-10900K CPU (3.70 GHz) and an NVIDIA GeForce RTX 3090 GPU with 24 GB of memory. The software environment was based on Ubuntu 22.04 and CUDA 11.8, with Python 3.9.23 and PyTorch 2.2.2 used for model construction and training. All experiments were conducted under the same computing environment to ensure the fairness and reproducibility of the experimental results. The offset parameters in DSConv were optimized using the same learning rate as the other model parameters, without separate learning-rate tuning. The training hyperparameters of the model are listed in Table 3.

### 2.6. Evaluation Indicators

To test the performance of the DWEC-YOLO model, we use Precision (P), Recall (R), F1-score, and mean Average Precision (mAP), where TP, FP, and FN represent true positives, false positives, and false negatives. Specifically, Precision reflects the percentage of correct predictions among all positive results to evaluate false alarms, while Recall measures how many real targets the model successfully finds to monitor missed detections. The F1-score takes the harmonic average of these two metrics to show their overall balance, and mAP@0.5 calculates the final average precision across all categories at an IoU threshold of 0.5. The exact calculation formulas are listed below:

(12) $$p\;=\;\frac{T_{p}}{T_{p}\;+\;F_{p}}$$

(13) $$R=\frac{T_{p}}{T_{p}+{\;F}_{n}}$$

(14) $$F_{1}=\frac{2\;\times \;P\;\times \;R}{P+R}$$

(15) $$mAP=\frac{1}{N}\int_{0}^{1}P\left(R\right)dR$$

To rigorously test localization quality, we also included mAP@0.5:0.95, which averages the AP values across IoU thresholds from 0.50 to 0.95 using a 0.05 step size. This strict metric provides a more comprehensive assessment of how well the model handles varying precision demands.

## 3. Results

### 3.1. Ablation Analysis and Module Validation

Ablation experiments were conducted to evaluate the effect of data augmentation and the individual and combined contributions of the modules incorporated into DWEC-YOLO. First, the effect of data augmentation was evaluated by comparing YOLO11 trained with and without augmentation under the same experimental settings. Each training configuration was repeated three times using random seeds of 0, 42, and 123, while the dataset split, training settings, and hardware configuration were kept identical. The results are reported as mean ± standard deviation (SD) in Table 4. As shown in Table 4, data augmentation improved Precision, Recall, F1-score, mAP@0.5, and mAP@0.5:0.95 compared with training without augmentation. These results demonstrate the positive effect of data augmentation on the overall detection performance of YOLO11.

Starting from the YOLO11 baseline, the C3k2-DySnake, EUCB-SC, CoordAtt, and Wise-ShapeIoU modules were evaluated individually and in different combinations to assess their effects on detection performance, model size, and computational complexity (FLOPs). In addition, the incremental contributions of the modules were analyzed using the configurations in Models A, D, G, and H. The results are reported as mean ± standard deviation (SD) in Table 5.

As shown in Table 5, different modules lead to varying changes in detection performance. Starting from the YOLO11 baseline, adding C3k2-DySnake (Model A) increases the mean mAP@0.5 from 95.29% to 95.51% and mAP@0.5:0.95 from 87.28% to 87.73%, corresponding to changes of 0.22 and 0.45 percentage points, respectively. With the further addition of EUCB-SC (Model D), mAP@0.5 and mAP@0.5:0.95 increase to 95.82% and 88.76%, respectively, corresponding to additional changes of 0.31 and 1.03 percentage points. After incorporating CoordAtt (Model G), mAP@0.5 changes slightly from 95.82% to 95.76%, while mAP@0.5:0.95 increases from 88.76% to 89.28%. Finally, adding Wise-ShapeIoU (Model H) increases mAP@0.5 from 95.76% to 97.55% and mAP@0.5:0.95 from 89.28% to 91.21%, corresponding to increases of 1.79 and 1.93 percentage points, respectively.

By incorporating shape-aware constraints and dynamically weighting regression samples, Wise-ShapeIoU considers both the geometric characteristics of the bounding boxes and the relative importance of different regression samples during localization. At the IoU = 0.50 threshold, improvements in box overlap can directly change whether predictions meet the matching criterion, making AP@0.5 sensitive to localization accuracy. In this experiment, the mean mAP@0.5 increased from 95.76% to 97.55%. In contrast, mAP@0.5:0.95 averages AP over IoU thresholds from 0.50 to 0.95, and the mean value increased from 89.28% to 91.21% across these thresholds. In group-housed pig scenes, posture variation and inter-pig overlap can make precise boundary alignment more challenging, which may make the shape-aware regression strategy particularly relevant to this task.

### 3.2. Generalization Performance of the Proposed Method on Advanced YOLO Models

To evaluate the generalization capability of the proposed DWEC method across different YOLO architectures, DWEC was extended to YOLOv12n, YOLOv13n, and YOLOv26n under the same dataset and experimental settings. During the adaptation, the DWEC components were incorporated into the corresponding stages of different YOLO architectures according to their original functions. Specifically, the C3k2-DySnake module was applied to enhance feature extraction, the EUCB-SC module was used to improve multi-scale feature fusion, and the CoordAtt mechanism was introduced for feature refinement. Meanwhile, Wise-ShapeIoU was directly adopted as the bounding box regression loss.

As shown in Table 6, the adapted DWEC variants consistently improved the detection performance of all evaluated architectures. For YOLOv12n, mAP@0.5 grew by 2.58 percentage points, whereas mAP@0.5:0.95 increased by 5.16 percentage points. For YOLOv13n, mAP@0.5 reached 97.66%, while mAP@0.5:0.95 reached 93.69%. Consistent improvements were also observed on YOLOv26n, where mAP@0.5 increased to 96.86% and mAP@0.5:0.95 reached 89.39%, both higher than those of the original YOLOv26n. Meanwhile, Precision, Recall, and F1-score also showed consistent improvements across all models. These results demonstrate the effectiveness and transferability of DWEC across different YOLO architectures.

### 3.3. Comparative Experiments

To demonstrate the competitive edge of the proposed DWEC-YOLO model in pig behavior recognition, comparative experiments are conducted against several mainstream lightweight object detection models, including YOLOv5n, YOLOv7-Tiny, YOLOv8n, YOLOv10n, YOLO11n, and YOLOv12n. In addition, the larger YOLO11s model is introduced as a same-family scaling baseline to investigate whether the performance improvements of DWEC-YOLO are mainly attributed to the proposed modules rather than increased model capacity. The cross-model evaluation focuses on primary accuracy indicators (Precision, Recall, F1-score, mAP@0.5, and mAP@0.5:0.95), while also considering efficiency parameters such as model size and computational complexity (FLOPs). All models are trained and tested under identical training and inference settings, and the results are reported in Table 7. Latency was measured on the NVIDIA GeForce RTX 3090 GPU described in Section 2.5, using a batch size of 1.

From the table, DWEC-YOLO achieves favorable performance across all evaluation metrics, achieving the highest Precision and Recall while maintaining a lightweight architecture. The model achieves an mAP@0.5 of 97.57%, representing an improvement of 2.65 percentage points over YOLO11n and remaining close to the performance of the larger YOLO11s model. For the stricter mAP@0.5:0.95 metric, DWEC-YOLO reaches 90.89%, improving YOLO11n by 4.13 percentage points and indicating enhanced localization capability under different IoU thresholds. Consistent improvements are also observed in Precision, Recall, and F1-score, demonstrating the ability of the proposed model to reduce false detections and missed targets. Although YOLOv8n and YOLOv12n achieve competitive results on certain metrics, DWEC-YOLO provides a favorable balance between detection accuracy and model complexity.

DWEC-YOLO boosts detection accuracy while maintaining a compact model structure. Compared with YOLO11n, DWEC-YOLO has a slightly larger model size (5.9 MB vs. 5.2 MB), parameter count (2.9 M vs. 2.6 M), and computational load (7.1 vs. 6.3 GFLOPs), as well as higher inference latency (4.3 vs. 1.4 ms), while achieving higher detection accuracy across the main evaluation metrics. Compared with the larger YOLO11s model (18.3 MB, 9.4 M parameters, and 21.3 GFLOPs), DWEC-YOLO maintains competitive detection accuracy with much lower model size, parameter count, and computational complexity. These results indicate that the performance gains of DWEC-YOLO are not simply attributed to increased model capacity, but mainly benefit from the proposed architectural enhancements.

The measured inference latency of 4.3 ms/frame corresponds to a theoretical processing rate of approximately 232.6 frames/s, indicating that the proposed model has sufficient inference speed for real-time frame-level behavior detection under the tested hardware conditions. This assessment is based on model inference latency, while the overall processing speed in practical deployment may also depend on image acquisition, preprocessing, and post-processing.

### 3.4. Per-Class Performance, Confusion Analysis, and Occlusion Robustness

Given the imbalanced distribution of behavior categories, overall detection metrics may not fully reflect the performance of minority behavior categories. Therefore, the precision, recall, and AP@0.5 of DWEC-YOLO were evaluated across individual behavior categories. The results are presented in Table 8.

As shown in Table 8, the detection performance varies across behavior categories, with AP@0.5 ranging from 93.28% to 99.35%. Despite the substantial difference in the number of instances among categories, the three aggressive behaviors achieve relatively high AP@0.5 values, reaching 99.35% for Fighting, 96.36% for Ear Biting, and 99.16% for Tail Biting. This indicates that the proposed model maintains effective detection performance for the minority aggressive behaviors despite the imbalanced class distribution.

**Figure 9:**
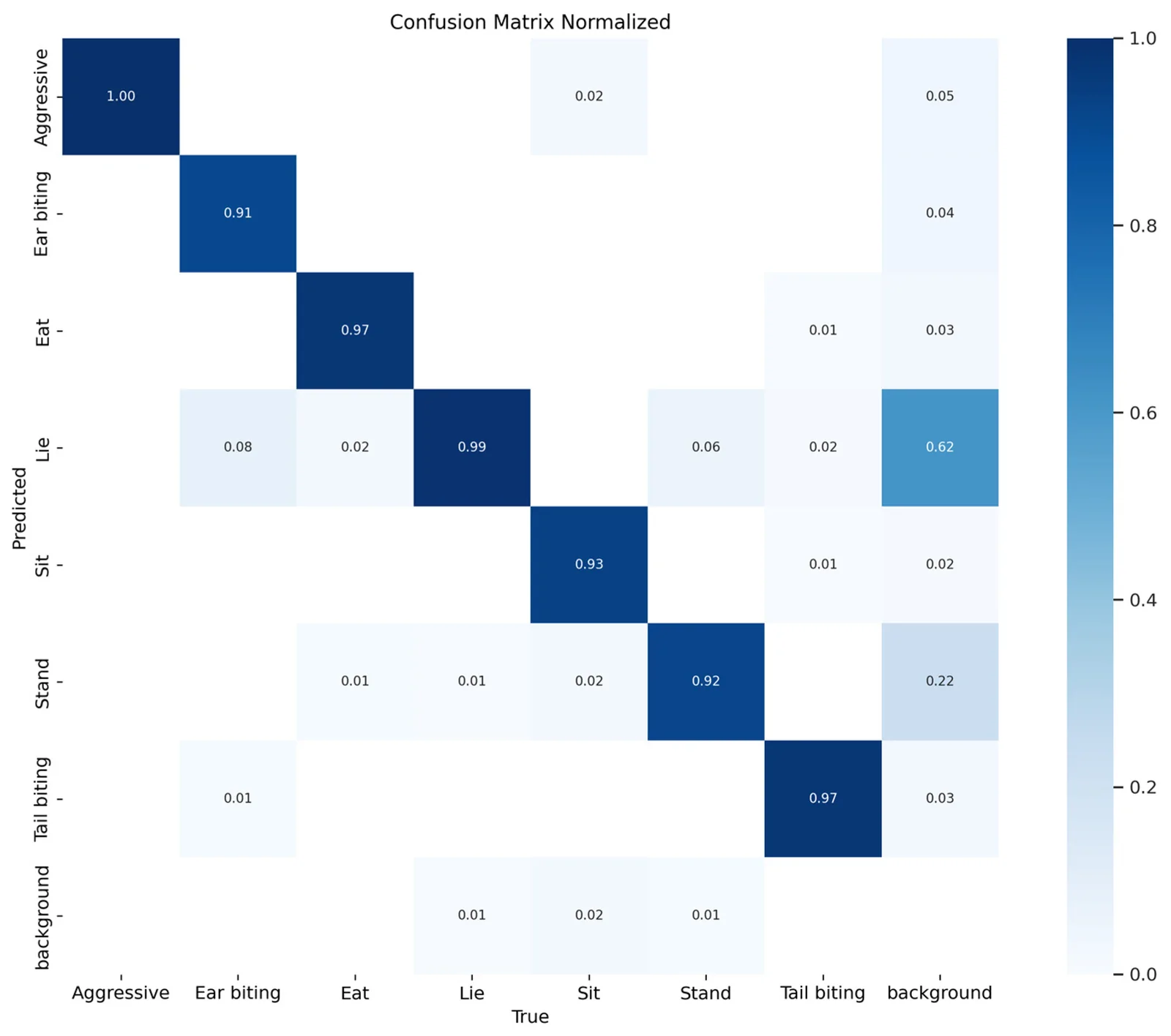
Normalized confusion matrix.

To further examine the class-specific prediction patterns of DWEC-YOLO, a normalized confusion matrix was generated on the test set, as shown in Figure 9. The diagonal entries represent the proportions of instances correctly predicted for each behavior category. Overall, relatively high correct-prediction rates were observed across all seven behavior categories, including the minority aggressive behaviors. The main inter-class confusions occurred between visually similar behaviors, particularly Ear Biting and Lie (8%) and Stand and Lie (6%). In addition, background regions were mainly misclassified as Lie (62%) and Stand (22%).

These results indicate that DWEC-YOLO maintained relatively consistent detection performance across the seven behavior categories, although some misclassifications were observed between visually similar behaviors. Beyond class-specific performance, the robustness of DWEC-YOLO to partial occlusion was further assessed by dividing the test instances into occluded and unoccluded groups according to the manual occlusion annotations. At an IoU threshold of 0.5, the model achieved mAP@0.5 values of 96.19% for unoccluded instances and 93.01% for occluded instances. Despite this decrease of 3.18 percentage points, the model maintained relatively high detection performance for occluded instances, providing quantitative evidence of its robustness to partial occlusion.

### 3.5. Visualization Results and Analysis

To provide a visual comparison of model performance in complex behavioral scenarios, predictions from both the baseline YOLO11 and our DWEC-YOLO on the test set were visualized and contrasted.

**Figure 10:**
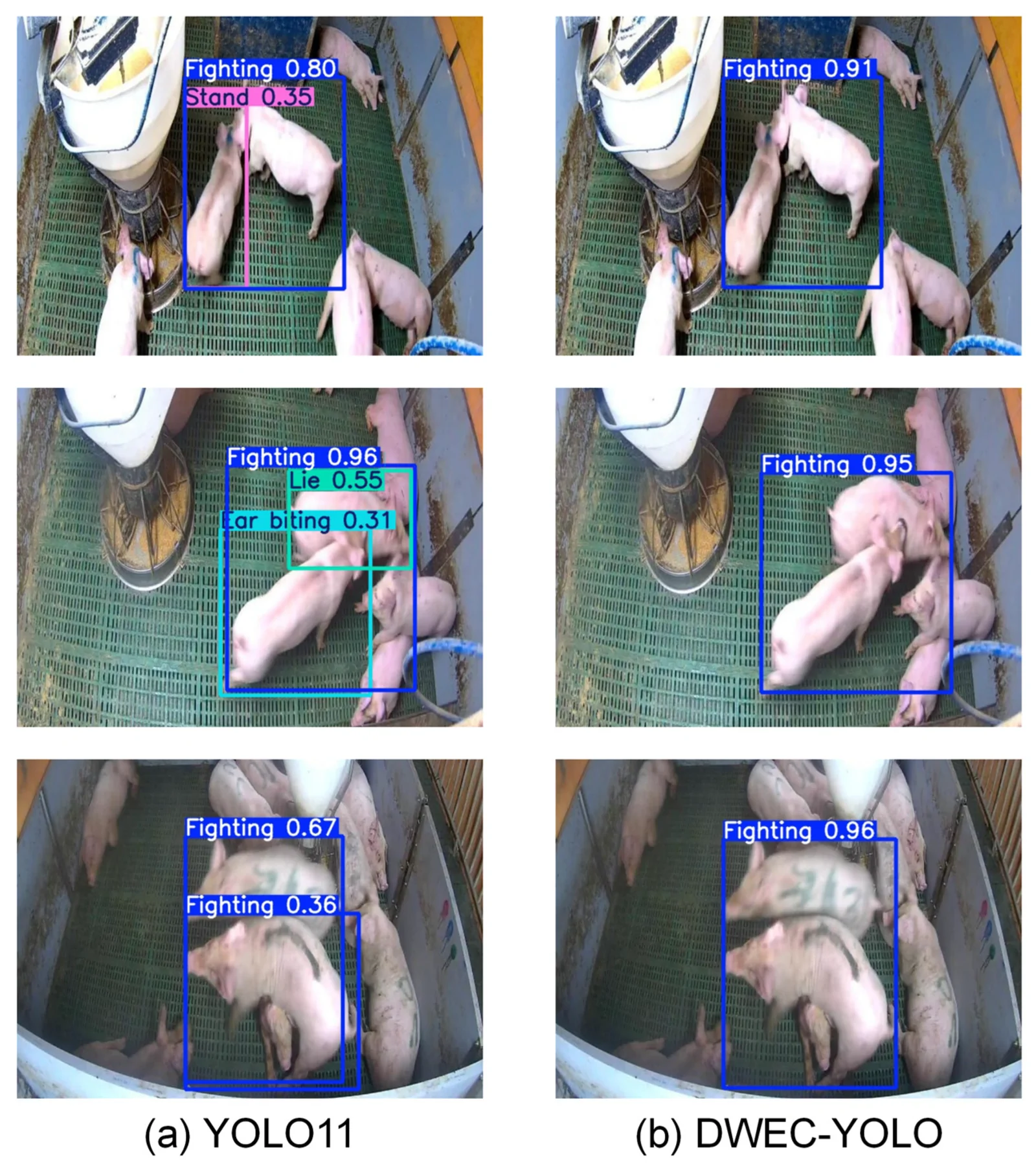
Comparison of fighting behavior detection results. (**a**) YOLO11. (**b**) DWEC-YOLO.

In fighting scenarios, the behavior is defined by direct physical interactions between pigs rather than by the actions of individual animals. As shown in Figure 10a, the baseline YOLO11 can detect the interaction region but assigns separate behavior labels to the pigs involved. For example, some pigs are classified as stand while others are classified as lie, resulting in inconsistent behavioral predictions within the same scene. In contrast, Figure 10b shows that DWEC-YOLO consistently identifies the interaction as fighting rather than assigning different posture labels to pigs involved in the same aggressive event. This yields more coherent detection results and reduces redundant predictions.

**Figure 11:**
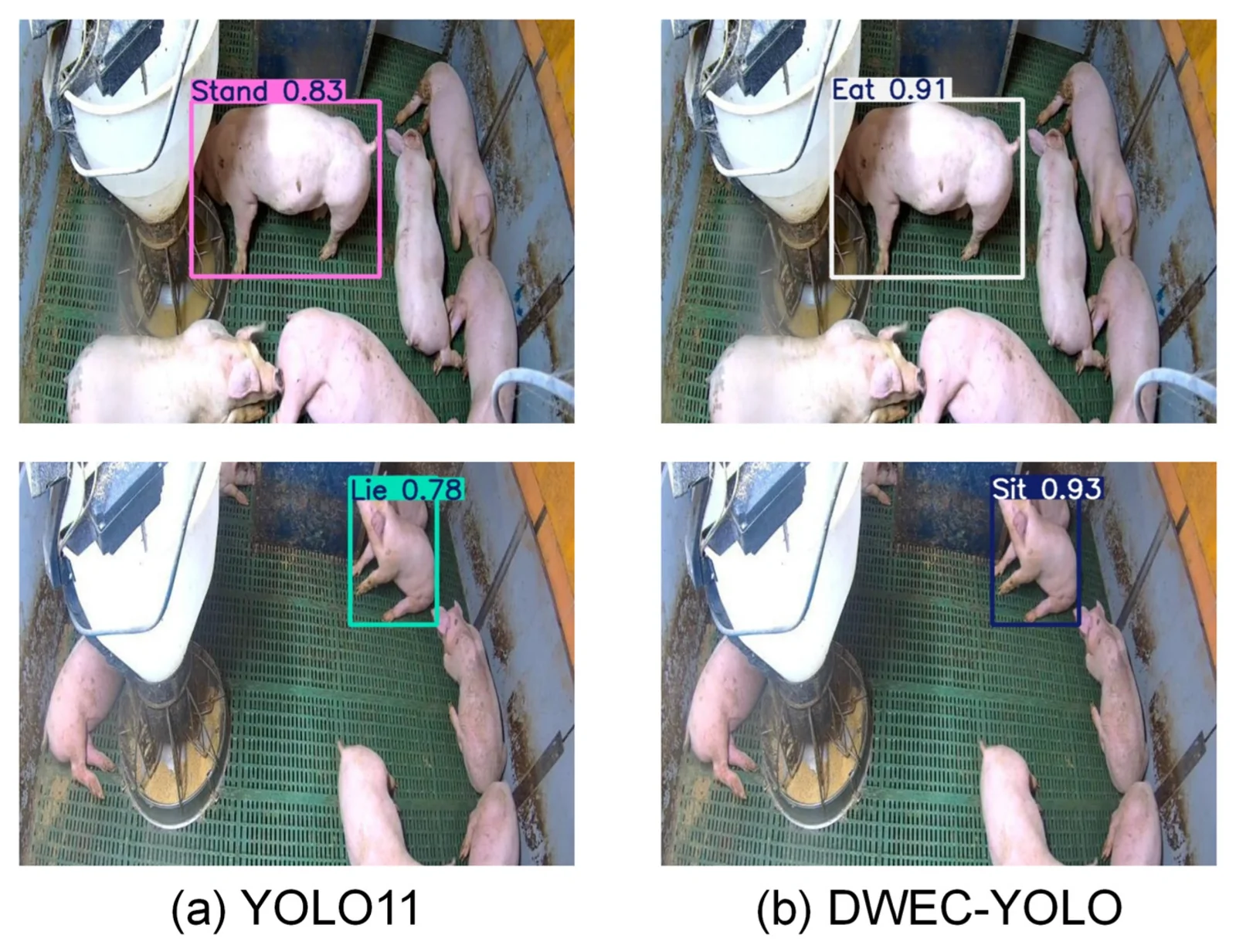
Comparison of static behavior detection results. (**a**) YOLO11. (**b**) DWEC-YOLO.

For static pig behaviors, the high similarity in posture and body structure often leads to category confusion during prediction. As shown in Figure 11a, the baseline YOLO11 misclassifies feed as stand in some cases and also exhibits confusion between the sit and lie categories. In contrast, as shown in Figure 11b, DWEC-YOLO distinguishes these visually similar behaviors more accurately, with the predicted bounding boxes closely matching the ground-truth annotations. These examples indicate its improved capability for fine-grained behavior discrimination.

**Figure 12:**
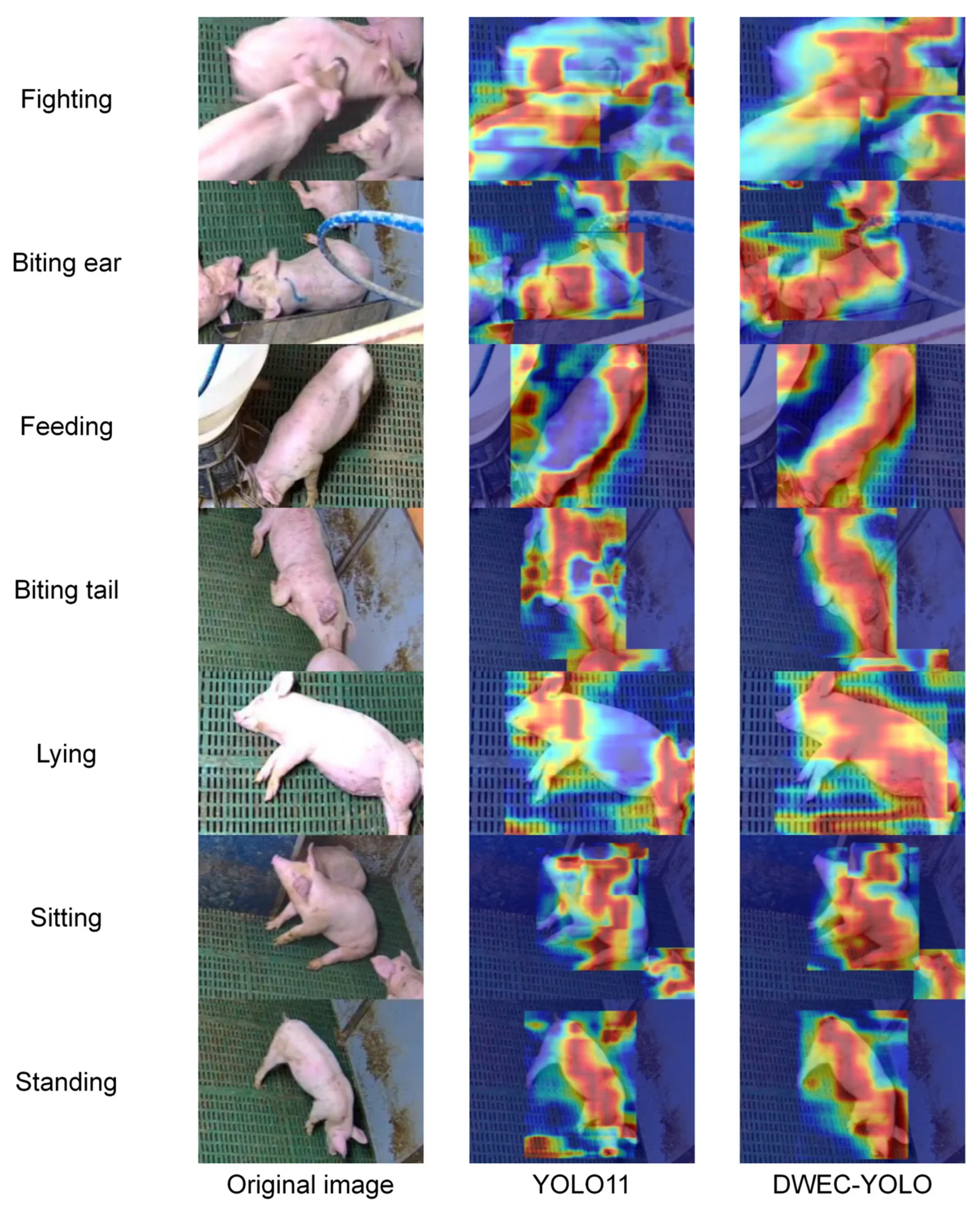
Comparative Grad-CAM++ visualizations of YOLO11 and DWEC-YOLO for different pig behavior categories.

To further investigate the feature attention patterns of the models, Grad-CAM++ [46] was employed to visualize the prediction process. Figure 12 presents a comparison between YOLO11 and DWEC-YOLO across different behavior categories. In interaction behaviors such as fighting, ear biting, and tail biting, YOLO11 exhibits dispersed activations and allocates part of its attention to background regions. By contrast, the activation regions of DWEC-YOLO are primarily concentrated on the key interaction areas between pigs, particularly around the head, neck, and tail. Similar differences can also be observed in individual behaviors, including sit, feed, stand, and lie. DWEC-YOLO consistently focuses on behavior-related regions, such as body posture, head position, and the contact area between the body and the ground, whereas YOLO11 still shows attention shifts and background interference in some samples.

The Grad-CAM++ maps reveal that DWEC-YOLO channels its focus more accurately and consistently onto behavior-relevant regions, enabling more effective feature extraction for both interaction and individual behaviors. To quantitatively support this observation, the Feed behavior was further analyzed by comparing the mean CAM activation within the target pig region with that in the background and pen furniture. The mean CAM activation was 0.4487 for the target pig region, compared with 0.2811 ± 0.1307 for the background and pen furniture. This higher activation within the target pig region indicates that DWEC-YOLO primarily focuses on pig-related visual features rather than surrounding pen furniture when recognizing the Feed behavior. These findings provide quantitative support for the more focused activation pattern of DWEC-YOLO and its superior recognition performance compared with YOLO11.

## 4. Discussion

Animal behavior recognition performance depends not only on model architecture but also on the composition of the dataset, particularly the coverage of behavior categories and the diversity of scenarios. However, tracking the full spectrum of livestock behavior remains difficult because gathering diverse video footage and completing exhaustive labeling demand immense effort. Table 9 summarizes recent livestock behavior recognition studies in terms of animal species, detection models, and behavior categories. Evidently, the imbalance in research focus between routine and aggressive behaviors restricts the recognition of abnormal behaviors in group-housing environments. Accordingly, this study expands behavior coverage by introducing a comprehensive dataset, which encompasses four routine postures (standing, lying, feeding, and sitting) as well as three aggressive actions (fighting, ear biting, and tail biting), thereby supporting multi-behavior recognition in complex pig farming environments.

Beyond the limited coverage of behavior categories, pig behavior recognition in group-housing environments remains challenging due to frequent occlusion, overlap, and close interactions between individuals, which can hinder the extraction of discriminative behavioral features. In addition, visually similar behaviors and posture transitions may increase the difficulty of behavior discrimination from individual images. These challenges highlight the need for more effective feature extraction and multi-scale feature fusion to improve recognition performance in complex farming environments.

Table 10 places DWEC-YOLO alongside representative animal behavior recognition methods, including those based on spatiotemporal Transformers, skeleton keypoints, and 3D convolutional networks. While these alternative frameworks achieve high accuracy in specific tasks, their reliance on continuous video sequences or temporal information renders them highly vulnerable to the frequent occlusions and social interactions typical of group-housing environments. Furthermore, such temporal modeling inevitably escalates computational complexity and deployment costs, thereby limiting their applicability in resource-constrained, real-world farming scenarios. In contrast, DWEC-YOLO is based on a one-stage detection framework and directly processes raw images for multi-behavior recognition. This reduces the dependence on temporal information and enhances its suitability for practical deployment while maintaining competitive detection performance.

In summary, while our findings validate the strength of DWEC-YOLO, a few remaining bottlenecks deserve attention. First, aggressive behavior samples remain relatively limited in the dataset and class imbalance still exists, which may affect the recognition stability of infrequent behaviors. Second, this work performs behavioral parsing using independent images without fully utilizing temporal information. Each image was annotated independently based on the behavior clearly observable in that frame, without a predefined minimum duration threshold. Thus, single-frame annotations cannot distinguish momentary contact from sustained biting based on temporal continuity alone. Incorporating temporal information in future work may help characterize the duration and continuity of such behaviors. Third, our data collection was conducted on a single commercial farm, meaning that variations in interior layouts, lighting conditions, and camera viewpoints across different facilities may constrain the network’s adaptability. Fourth, temperature, relative humidity, and other microclimatic parameters were not systematically recorded during data collection, and the public dataset did not provide such information either. Therefore, the robustness of the proposed system under different climatic conditions, particularly under extreme high or low temperatures, could not be directly assessed in the present study. In addition, the long-term durability of the sensors and the overall system under highly humid barn conditions was not evaluated in the present study. The service life of the equipment under prolonged high-humidity exposure remains to be determined through long-term field testing, including evaluation of its durability and maintenance requirements. To address these shortcomings, upcoming research intends to broaden the dataset’s scope across multiple livestock facilities and climatic conditions and explore cross-environment training strategies to enhance generalization. Multi-object tracking and temporal smoothing will also be explored to reduce frame-level prediction jitter and occasional false detections in continuous video streams, while long-term field evaluation under different climatic conditions will be conducted to further assess the robustness and practical applicability of the proposed system.

## 5. Conclusions

Recognizing normal and aggressive behaviors in group-housed pigs is essential for monitoring their health, detecting anomalies, and improving farm management. To address the limited focus on aggressive behaviors and high computational cost in existing methods, we built a multi-class pig behavior dataset including normal behaviors (standing, lying, feeding, sitting) and aggressive behaviors (fighting, ear biting, tail biting). Based on YOLO11, we proposed the DWEC-YOLO model for detecting multiple behaviors in complex group environments. Experiments using our dataset show that DWEC-YOLO achieved 96.17% Precision, 95.25% Recall, and 95.69% F1-score, with mAP@0.5 and mAP@0.5:0.95 of 97.57% and 90.89%, respectively. Compared with YOLO11, mAP@0.5 and mAP@0.5:0.95 increased by 2.65 and 4.13 percentage points, indicating the effectiveness of the proposed improvements in distinguishing both normal and aggressive behaviors under complex group-housing conditions.

Moreover, the lightweight (5.9 MB) and computationally efficient (7.1 GFLOPs) nature of DWEC-YOLO strikes a good balance between accuracy and computational cost, indicating its potential for practical livestock monitoring applications. In the future, combining data from multiple scenarios and modeling temporal behavior will help the model adapt easily to new environments and handle round-the-clock monitoring in busy commercial facilities.

## Figures and Tables

**Table 1:** Distribution of pig behavior instances across the training, validation, and testing sets.

Class	Train	Val	Test	Public Total	Farm Total	Overall Total
Lying	8369	2420	1161	7361	4589	11950
Standing	1310	334	185	1127	702	1829
Sitting	312	89	44	281	164	445
Feeding	528	144	75	460	287	747
Fighting	334	92	47	291	182	473
Ear Biting	487	132	69	424	264	688
Tail Biting	547	175	77	492	307	799

**Table 2:** Behavior recognition rules for pigs.

Class	Description	Instance
Lying	The pig lies in a lateral or sternal position with minimal movement.	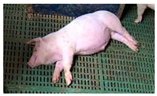
Standing	The pig supports its body weight on all four limbs while exhibiting no obvious displacement.	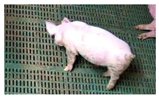
Sitting	The pig rests on hind limbs with forelimbs supporting the upper body.	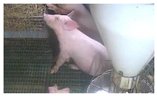
Feeding	The pig feeds with its head near the feeder continuously.	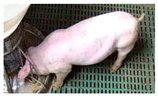
Fighting	The pig engages in physical interaction with other pigs involving collisions, head knocking or biting.	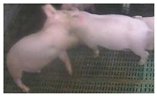
Ear Biting	The pig bites or pulls the ear of another pig as a form of social aggression.	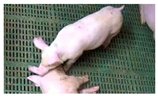
Tail Biting	The pig bites or pulls the tail of another pig during social or aggressive interaction.	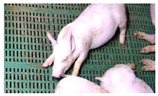

**Table 3:** DWEC-YOLO training parameters.

Configuration Item	Value
Input image size	640 × 640
Optimizer	Stochastic Gradient Descent (SGD)
Initial learning rate	0.01
Momentum	0.937
Weight decay	0.0005
Batch size	16
Epochs	200

**Table 4:** Effect of data augmentation on YOLO11.

Training Setting	Precision (%)	Recall (%)	F1-Score (%)	mAP@0.5 (%)	mAP@0.5:0.95 (%)
YOLO11 without augmentation	91.67 ± 0.87	85.72 ± 0.74	88.26 ± 0.38	91.44 ± 0.47	81.44 ± 0.37
YOLO11 with augmentation	92.07 ± 1.03	91.52 ± 1.62	91.73 ± 0.43	95.29 ± 0.43	87.28 ± 0.48

**Table 5:** Ablation study of the proposed DWEC-YOLO on the testing set.

Model	C3k2- DySnake	EUCB-SC	CoordAtt	Wise- ShapeIoU	Precision (%)	Recall (%)	F1-Score (%)	mAP@0.5 (%)	mAP@0.5:0.95 (%)	Size (MB)	FLOPs (G)
YOLO11	×	×	×	×	92.07 ± 1.03	91.52 ± 1.62	91.73 ± 0.43	95.29 ± 0.43	87.28 ± 0.48	5.2	6.3
A	√	×	×	×	93.60 ± 1.01	91.13 ± 2.28	92.30 ± 0.68	95.51 ± 0.31	87.73 ± 0.21	5.7	6.7
B	×	√	×	×	92.66 ± 0.84	91.03 ± 1.35	91.81 ± 0.48	95.07 ± 0.34	87.60 ± 0.61	5.4	6.8
C	×	×	√	×	93.99 ± 0.45	91.21 ± 1.48	92.55 ± 0.57	95.39 ± 0.43	88.35 ± 0.76	5.3	6.3
D	√	√	×	×	94.21 ± 0.45	91.48 ± 0.94	92.80 ± 0.69	95.82 ± 0.43	88.76 ± 0.53	5.8	7.1
E	√	×	√	×	92.78 ± 1.27	91.91 ± 0.76	92.30 ± 0.31	95.92 ± 0.39	88.45 ± 0.39	5.7	6.7
F	×	√	√	×	94.10 ± 0.09	91.48 ± 1.49	92.73 ± 0.81	95.75 ± 0.51	88.58 ± 0.61	5.4	6.8
G	√	√	√	×	94.73 ± 0.04	91.30 ± 2.64	92.94 ± 1.40	95.76 ± 0.80	89.28 ± 1.29	5.9	7.1
H	√	√	√	√	96.03 ± 0.68	95.39 ± 0.44	95.69 ± 0.13	97.55 ± 0.65	91.21 ± 0.47	5.9	7.1

**Table 6:** Performance comparison of the proposed method with recent YOLO models on the testing set.

Model	Precision (%)	Recall (%)	F1-Score (%)	mAP@0.5 (%)	mAP@0.5:0.95 (%)
YOLO11n	91.89	90.79	91.33	94.92	86.76
DWEC-YOLO(v11)	96.17	95.25	95.69	97.57	90.89
YOLOv12n	94.03	89.25	91.53	94.57	87.81
DWEC-YOLO(v12)	95.28	96.35	95.79	97.15	92.97
YOLOv13n	91.95	91.61	91.77	94.34	87.03
DWEC-YOLO(v13)	96.64	96.24	96.42	97.66	93.69
YOLOv26n	92.81	89.41	91.07	93.44	85.49
DWEC-YOLO(v26)	94.77	94.73	94.73	96.86	89.39

**Table 7:** Behavior recognition performance of different models on the testing set.

Model	Precision (%)	Recall (%)	F1-Score (%)	mAP@0.5 (%)	mAP@0.5:0.95 (%)	Size (MB)	Params (M)	FLOPs (G)	Latency (ms)
YOLOv5n	92.02	88.05	89.87	93.80	80.03	7.1	1.8	4.2	1.2
YOLOv7-Tiny	91.70	89.96	90.82	94.07	81.88	11.8	6.0	6.5	1.6
YOLOv8n	93.21	89.65	91.35	94.98	86.33	6.0	3.0	8.1	2.4
YOLOv10n	92.67	88.00	90.15	93.37	86.88	5.5	2.3	6.5	2.2
YOLOv12n	94.03	89.25	91.53	94.57	87.81	5.3	2.6	6.3	2.0
YOLO11n	91.89	90.79	91.33	94.92	86.76	5.2	2.6	6.3	1.4
YOLO11s	96.10	93.34	94.70	97.93	93.37	18.3	9.4	21.3	3.1
DWEC-YOLO	96.17	95.25	95.69	97.57	90.89	5.9	2.9	7.1	4.3

**Table 8:** Per-class detection performance of DWEC-YOLO on the testing set.

Class	Precision (%)	Recall (%)	AP@0.5 (%)
Lying	97.62	97.14	98.77
Standing	94.04	90.91	97.21
Sitting	91.00	90.70	93.28
Feeding	98.02	98.04	98.87
Fighting	97.37	100.00	99.35
Ear Biting	97.13	91.46	96.36
Tail Biting	98.03	98.48	99.16

**Table 9:** Comparison of behavior category coverage in recent livestock behavior recognition studies.

Species	Study	Model	Behavior Category
Pig	Zeng et al. [47]	Enhanced YOLOv8n (MACA-Net)	Feeding, sternal lying, lateral lying, and standing
Pig	Sukkuea et al. [48]	Improved YOLOv8n (YOLOv8ma)	Drinking, feeding, sleeping, and standing
Pig	Pu et al. [49]	Improved YOLOv8n	Standing, sternal lying, lateral lying, and feeding
Sheep	Hu et al. [50]	Improved YOLOv5	Standing, lying, and walking
Pig	Zhang et al. [51]	SSD+MobileNet (SBDA-DL)	Drinking, urination, and mounting
Cattle	Long et al. [52]	Enhanced YOLO11 (FSCA-YOLO)	Feeding, drinking, standing, and lying
Cattle	Yu et al. [53]	Improved YOLOv5 (Res-DenseYOLO)	Drinking, feeding, lying, and standing

**Table 10:** Comparison of representative animal behavior recognition methods in terms of performance and applicability in farming scenarios.

Study	Model	Application Domain	Performance	Applicability in Farming Scenarios
Hu et al. [54] (2025)	PB-STR: Spatiotemporal Transformer	Pig multi-behavior recognition	mAP = 94.2%	Requires video sequences; computationally intensive
Hua et al. [55] (2023)	PoseC3D	Dairy cattle behavior recognition	Accuracy ≈ 92.6%	Relies on skeleton extraction; sensitive to occlusion
Pu et al. [56] (2025)	MobileNetV2-Autoformer	Pig aggressive behavior recognition	Accuracy ≈ 96.2%	Requires continuous frame input; designed for a single behavior
Shi et al. [57] (2025)	Temporal-Boost 3D CNN	Sheep tail-wagging recognition	Accuracy ≈ 96.2%	Requires temporal sequences; high computational cost
This study	DWEC-YOLO	Pig multi-behavior recognition	P = 96.17%, R = 95.25%, mAP@0.5 = 97.57%	Processes raw images directly

## Data Availability

The source code and trained weights of DWEC-YOLO are publicly available in the GitHub repository: https://github.com/wjie13114/DWEC-YOLO (version v1.0, accessed on 1 October 2026). The public dataset used in this study is available from Roboflow Universe at https://universe.roboflow.com/agonistic-behavior/agonistic-behavior-gs13j/dataset/1 (accessed on 17 February 2026). The self-collected dataset was obtained from a commercial pig farm in collaboration with Wens Foodstuff Group. Due to data ownership and privacy restrictions, the raw images are not publicly available. Researchers interested in accessing the self-collected dataset may contact the corresponding author. Data access will be subject to approval from the data provider.
